# Our problems and observations in 3D facial implant planning

**DOI:** 10.1186/s40902-022-00362-6

**Published:** 2022-10-12

**Authors:** Gianmarco Saponaro, Chiara Paolantonio, Giorgio Barbera, Enrico Foresta, Giulio Gasparini, Alessandro Moro

**Affiliations:** Maxillo-Facial Surgery Unit, IRCSS Fondazione Policlinico Universitario “A. Gemelli” – Università Cattolica del Sacro Cuore sede di Roma, Largo A. Gemelli 3, 00168 Rome, Italy

**Keywords:** PEEK, CAD-CAM, Custom implants, Prosthesis, 3D reconstruction, PEEK implants, Reconstruction, Patient-specific implants

## Abstract

**Background:**

Three-dimensional renderings of two-dimensional computed tomography data have allowed for more precise analysis in the craniofacial field. Design, engineering, architecture, and other industries have paved the way for the manipulation and printing of three-dimensional objects.

The usual planning is only carried out based on the bony structures, often without taking into consideration the presence of soft tissues and soft structures. During our practice, we have found ourselves facing the challenge posed by these structures; the aim of this article is to discuss our experience in designing implants presenting our tips and tricks for a better planning leading to an easy and reliable positioning.

**Case presentation:**

We have retrieved all patients in 5 years among those who underwent computer-aided design/computer-aided manufacturing implant placement in the last 5 years in order to review the eventual problems and the solutions found.

A total number of 25 patients were retrieved and, among them, 10 patients were selected, in which planning inaccuracy caused difficulties during implant placement and which then led to induced changes during the planning of similar cases or in which the problems were noted before or during the planning which led to changes in the plan to address those problems.

Six of the selected cases were polyetheretherketone facial implants for the correction of residual deformities in malformed or deformed patients.

One case was a delayed orbital reconstruction with a titanium implant.

Two cases were titanium functional and anatomical reconstruction of the mandible in patients with failed post-oncological reconstructions.

There was 1 case with a mandibular ramus complex and hard-to-treat fracture.

**Conclusions:**

The planning of the implant mostly relies on hard tissue three-dimensional reconstruction, but it should not be limited at what is immediately evident. A surgeon’s clinical experience should always guide the process, with knowledge of the patient’s anatomy and evaluation of the quality and of the soft tissue response being taken into consideration. The implant should always be tailored not only based on the bone defect and evaluations but also using the patient’s previewed and actual anatomy, evaluating eventual interferences and pitfalls.

## Background

Craniomaxillofacial surgery requires restitution of form and function and the correction of dysmorphology, disease, and defect. Soft tissue and osseous reconstructions are performed often in concert. The craniofacial region is complex, with topographic relations and compartments between anatomic sites. A comprehensive understanding of the problem is the first step before carrying out the definitive reconstruction.

Three-dimensional (3D) renderings of bi-dimensional (2D) computed tomography (CT) data have allowed for more precise analysis in the craniofacial field. Design, engineering, architecture, and other industries have paved the way for the manipulation and printing of 3D objects. Virtual surgical 3D simulation and planning have gained steam during the past several decades. It must be stressed that despite the many advantages of this technology, it cannot replace a surgeon’s clinical judgement or technical skill [1].

A 3D rendering of bone and soft tissue allows for in-depth consideration of the anatomy and problems at hand. Typically, the CT data are uploaded into a virtual surgical platform and manipulated in digital space. The region of interest can be viewed at scale or magnified and rotated to visualise contours and relations. Anatomy that will not be involved in treatment can be digitally removed to enhance the focus of the planned surgical site. Layers of structure can be virtually subtracted to allow for unfettered visualisation from multiple vantage points. Tools allowing linear, angular, and volumetric measurements can be used. These quantitative and morphologic values can be compared with “normal” or idealised situations to better understand what needs correction and establish the groundwork to develop a plan. The analysis is perhaps the most critical step in planning surgery. This is, in essence, the diagnostic phase: a descriptive morphologic understanding and capture of the problem. Analysis can be performed as follows: (1) defect (missing tissue), (2) deformity or dysmorphology (structures are present but are too small, large, or deformed), (3) malrelations (the anatomy is there but in the wrong place), or (4) some combination of these [2].

By following these steps, an appropriately sized, shaped, and volumed implant can be planned, but there is one last very important thing that needs to be addressed. The usual planning is only carried out based on the bony structures, often without taking into consideration the presence of soft tissues and soft structures. During our practice, we have found ourselves facing the challenge posed by these structures; the aim of this article is to discuss our experience in designing implants presenting our tips and tricks for a better planning leading to an easy and reliable positioning

First of all, soft tissues surrounding the bony defect must be considered, since they can hinder or act as a limitation to the insertion and positioning of the prosthesis.

The possible administration site should also be evaluated, with respect to the anatomy, function, and aesthetics.

We have decided to analyse the cases treated in the last 5 years with customised implants and select the most interesting ones, with regard to errors and tips in planning for easy and reliable placement, avoidance of important structures (e.g. tendons, nerves…), and surgical wounds.

## Case presentation

We have retrieved all patients among those who underwent computer-aided design/computer-aided manufacturing (CAD/CAM) implant placement in the last 5 years in order to review the eventual problems and the found solutions.

A total number of 25 patients were considered; among them, 10 patients were selected, in which planning inaccuracy caused difficulties during implant placement and which then led to induced changes during the planning of similar cases or in which the problems were noted before or during the planning which led to changes in the plan in order to address those problems.

Six among the selected cases were patients with congenital, developmental, or acquired malformations who had polyetheretherketone (PEEK) prostheses implanted to further correct some residual anomalies after former surgeries.

One case was represented by a late treatment for a medial orbital wall fracture.

Two cases were offered anatomical, functional mandibular reconstructions by means of titanium prostheses: in both cases, the patients had come to our attention presenting outcomes of failed mandibular reconstructions after resections performed for oncological reasons.

**Fig. 1:**
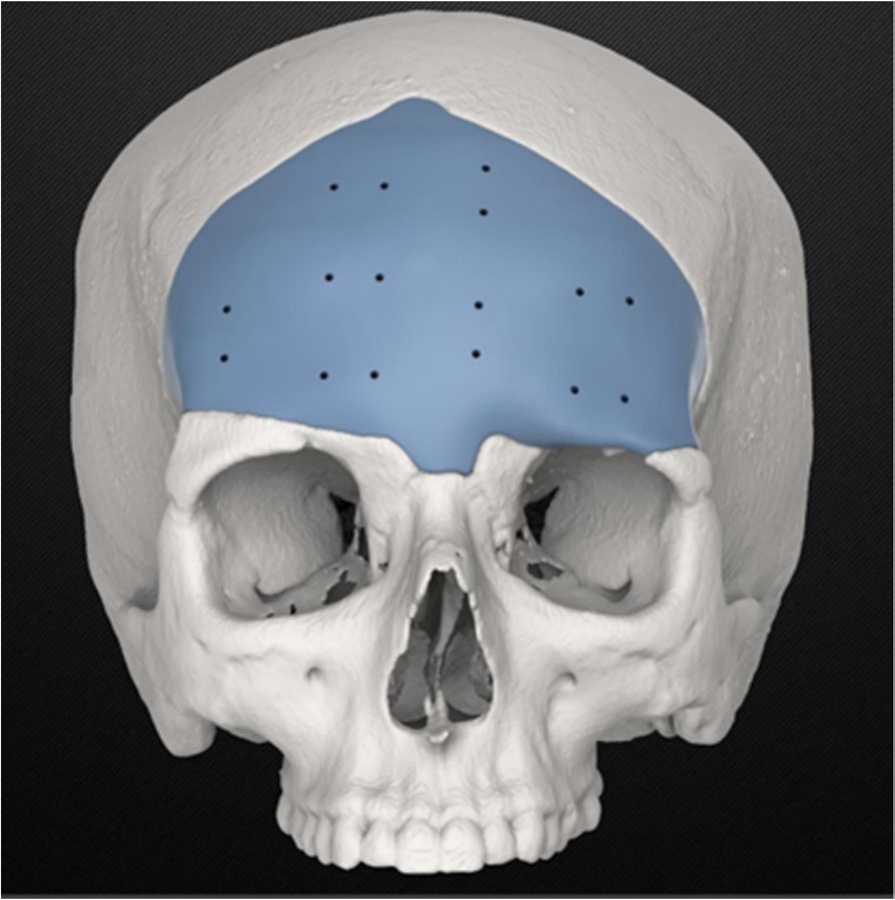
Case 5 3D render, the implant was planned as one piece partial onlay and partial full thickness; the problem encountered was that the implant was a little too big for the void, probably because some fibrotic parts were not visible in the CT; in addition, the thickness of the implant was exactly the same as the actual calvarial bone, and this has led to an increased rate of impingement with the fibrotic tissue, reducing the available space on the intracranial side

**Fig. 2:**
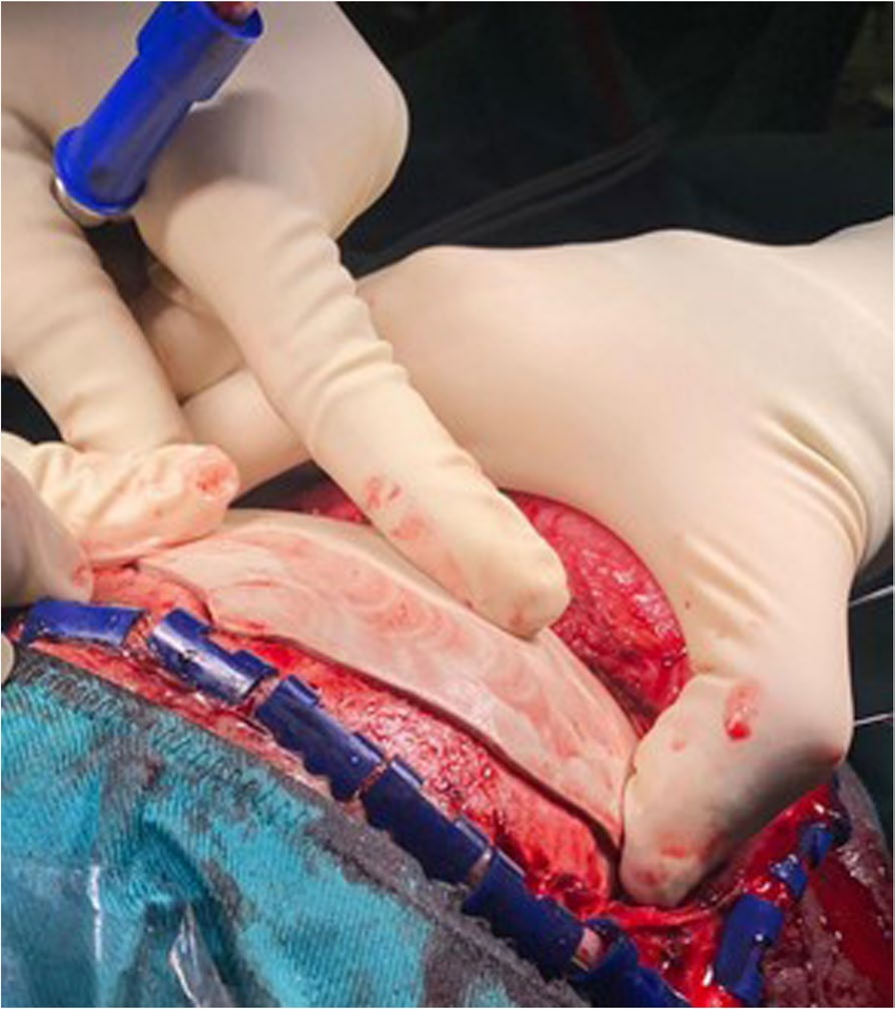
Intraoperatory view of case 5: the fitting problem is evident on the bone-implant inlay interface which creates an impingement that prevents the correct position

**Fig. 3:**
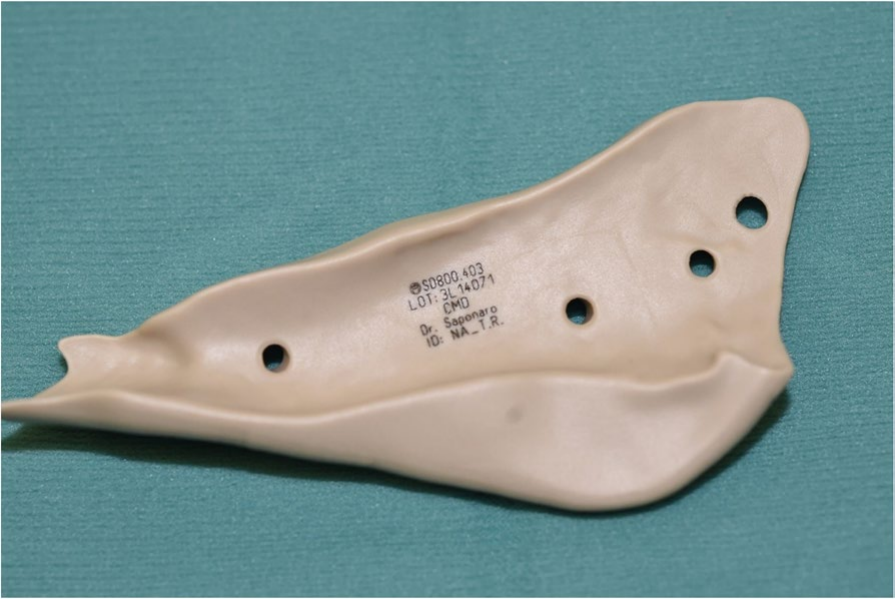
Case 1: vision of the lower mandibular groove in a PEEK implant; in this case, the implant was planned with an insufficient groove which provided insufficient retention and stability leading to difficulties in the correct positioning of the implant

**Fig. 4:**
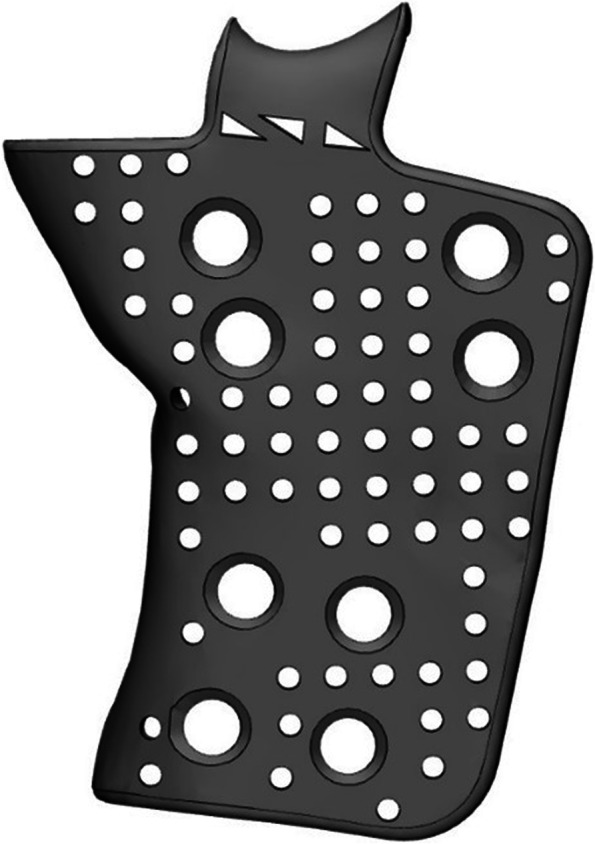
Case 10 – a titanium-reinforced plate to stabilise a mandibular pathologic fracture; a detachable flange is designed at the upper aspect to match with the sigmoid notch as a reference

**Fig. 5:**
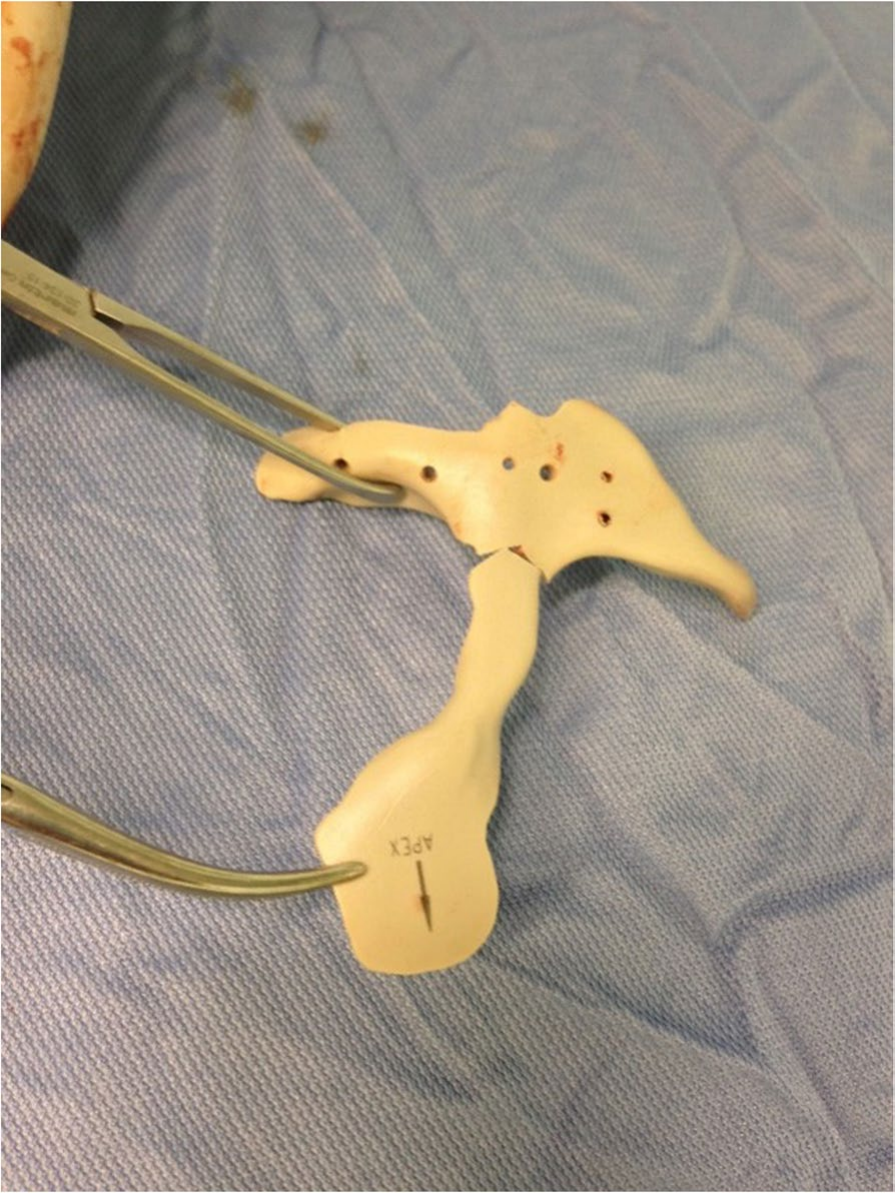
Zigomatic implant initially planned as one piece which was impossible to insert, the implant had then to be split in two parts intraoperatively

**Fig. 6:**
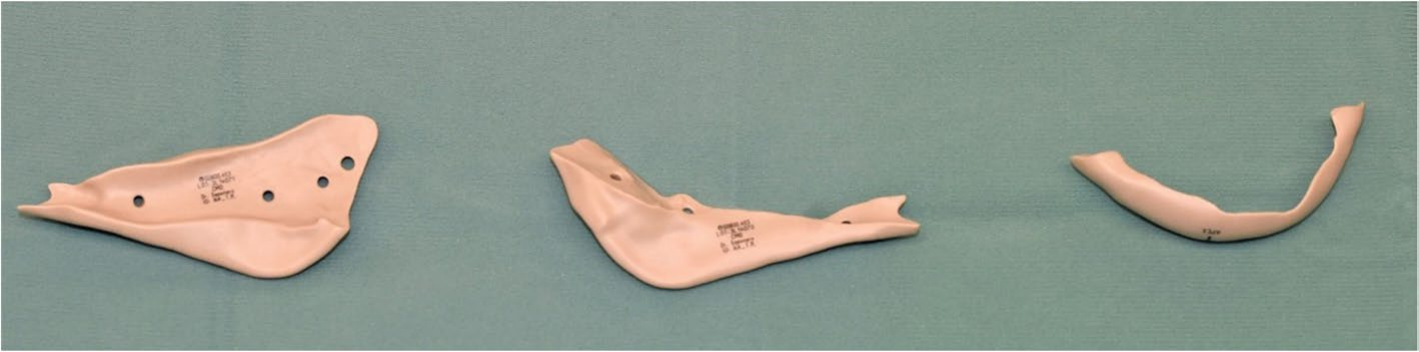
Three-piece implant designed for a full lower mandibular contour; the three implants have interlocking edges in order to provide the most reliable positioning possible and in order to avoid damage to the mental nerves during insertion

One case showed a complex, difficult-to-treat mandibular fracture affecting both the ramus and the body of the mandible (Figs. 1, 2, 3, 4, 5, and 6).

In case 1, there was a planning inaccuracy which had led to difficult stabilisation: this was a full lower jaw contour with PEEK implants, and the implants were divided into 3 pieces with reciprocal interdigitation in order to create 3-piece interlocking implants which were able to intersecate with one another for correct positioning and in order to avoid the mental foramina. In this patient, we experimented a planning inaccuracy characterised by a non-wrapping groove on the inferior border, which caused problems for primary retention and reliable placement. This problem has led to the request of a further wrapping groove in mandibular implants planned afterwards.

Case 2 showed another large mandibular angle/border prosthesisation.

In this patient, we have decided to create a 2-segment interlocking implant to avoid the mental nerve with a deep groove wrapping the mandible for an appropriate primary stability: indeed, the implant went almost autonomously into place and locked with the mandibular border. From our past experience, we had learned that it is always better to segment the implant into smaller implants avoiding the need of extended surgical wounds and reducing the risk of harming structures during insertion (e.g. mental nerve); also, a more accurate design and a more wrapping shape at the implant-bone interface are able to provide a more reliable positioning.

Case 3 presented an orbital medial wall delayed reconstruction.

The patient underwent reconstruction for unsatisfactory primary treatment. Originally, a one-piece titanium implant in the shape of mesh was planned; then, we realised that it would have been too big for positioning through a small incision and it would have impinged on the medial canthal tendon. For this reason, the planning was changed to a two-piece implant with two different plates to be inserted through two different cuts: one on the upper eyelid (Lynch’s fashion) and one in the subpalpebral/subciliar aspect. The two parts would have connected on the midline, creating a continuous plane [3].

Case 4 showed a zygomatic reconstruction in a patient affected by malar hypoplasia with neurofibromatosis I. The planned implant was a total zygomatic reconstruction to be inserted through one subpalpebral incision and one incision on the outer third of the eyebrow. Changes in the plan were made in order to allow the surgeon to place the implant through these two small incisions and a two-piece device with interlocking edges was planned: one for the zygomatic body and arch and one for the fronto-zygomatic buttress.

Case 5 showed an upper orbital ridge and frontal bone reconstruction secondary to facial-cranic trauma.

In this patient, the implant was mostly inlay, planned to fit inside the void left in the bone by cranial decompression.

The problem encountered in this case was that the implant exceeded the void of some millimetres, probably because some fibrotic parts were not visible in the CT; in addition, the thickness of the implant was exactly the same as the actual calvarial bone. This led to an increased rate of impingement with the fibrotic tissue, thereby further reducing the available space on the intracranial side.

Cases 6 and 7 were treated for different pathologies but with the same solutions: one was an onlay fronto-orbital reconstruction in a patient affected by Crouzon’s syndrome sequelae and one was a full-thickness fronto-orbital reconstruction after fronto-orbital removal due to osteomyelitis. In both patients, the reconstruction appeared easily feasible, but at a clinical evaluation, we realised that the skin would have been insufficient for quantity and elasticity in order to cover the implant; therefore, we decided to perform a 2-step surgery inserting a skin expander in order to increase the quantity and quality of the skin before placing the implant. The expanders were kept for 2 months and then removal with contemporary implant insertion was performed.

Cases 8 and 9 are similar cases: both were originally treated for oral cavity malignancies and underwent large mandibular resections. Reconstruction was immediately performed with fibula free flaps which failed in both cases. Both patients underwent radiotherapy with considerable scarring and retraction of the tissues. We decided to perform a secondary delayed reconstruction with CAD/CAM titanium mandible, but we realised that the retraction of the soft tissues would have made the insertion risky or impossible for insufficient wrapping of the implants. We decided to plan a multistep surgery postponing the implant insertion after 2 treatments with lipofilling in order to reconditionate the soft tissues and to have sufficient elasticity and coverage.

Case 10 was affected by a very severe class 2 dentoskeletal malformation, and he was treated through orthognathic surgery, but he then suffered from a pathological fracture.

He needed a reconstruction plate able to give enough strength to the residual mandible. Therefore, a large extended titanium implant was designed, in order to provide the best positioning.

A flange was extended to be positioned in the sigmoid notch as a reference, and the extension was made detachable in the eventual case of intraoperatory impingement.

## Discussion

Defects of the craniofacial complex may result in aesthetic deformities, functional damage, and psychological consequences [4]. Reconstruction with postoperative reestablishment of the contour and local shape has become a surgical priority [5]. The difficulties in the repair of the cranio-orbital region are related to the complex shape of such defects involving different curvature planes and different thicknesses. In cases necessitating resection of the orbital walls and rims, orbital reconstruction is advocated to avoid persistent alterations both aesthetical and functional [6, 7]. The perioperative shaping of autologous and/or alloplastic grafts is difficult and time consuming [8, 9].

Patient-specific implants (PSIs) have the advantage of being preoperatively designed to the exact shape and size of the defect, thus allowing a shortening of the operative time and a decrease in the number of intraoperative modifications and hence guaranteeing postoperative stability and good cosmetic results as well as avoiding donor site morbidity [10, 11].

The use of PSIs with specific digital designs is reportedly an effective technique for the treatment of craniofacial defects, reducing the need for manipulation in the intraoperative period and decreasing the overall surgery time [11–14]. Nevertheless, the accuracy of planning these kinds of implants is of vital importance, for a satisfactory outcome not only in terms of appropriateness of the reconstruction and corrections of the volumes, but also in terms of feasibility of the implantation without harming important structures and without the need of extended surgical approaches which, despite making the implant insertion possible, would cause aesthetical problems. Similarly to what was done for cases 1 to 4, the design should be made to minimise the surgical access and to avoid lesions to important structures. Segmentation of the implant provides a reduction of the volume for a single piece and makes insertion easier and less risky; of course, it should always be kept in mind that the pieces are all part of the same reconstruction, and the final outcome should reproduce the same outcome that a non-segmented implant would give. For this reason, it is always advisable to have some sort of interdigitation/interlocking parts that would serve as a reference for the appropriate reciprocal positioning of the segments [15, 16].

It is also noticeable how the interlock system can work as a litmus test for the correct positioning of the implants: an incorrect interdigitation between the segments is the callsign of an incorrect positioning. In those cases, the position should be reevaluated or, if the problem is found to be traced to the design of the prosthesis, then the surgeon should be able to face it by applying some impromptu solutions by drilling or cutting the implant until the best possible position is found.

In addition, an eventual error in the planning becomes maximised in one-piece implants; the implant can be seen as a lever, and in case of errors, the longer the lever, the bigger the error; subdividing the implant into different segments allows for redistribution of the error and makes the eventual correction easier by distributing the entity of eventual small, improvised corrections on the different segments.

Correct positioning is one of the factors that influence more the final appearance of the patient; the implants can be perfectly planned, but if perfect positioning is not achieved, the final result can be disappointing. For this reason, we suggest using all possible landmarks when designing the implant, using notches, foramina, etc. as reference points, and providing as many extensions as possible; the reference extensions can be also made detachable like in case 10 [16, 17].

In the case of lower mandibular border prosthesisation, a direct intraoperative visual check can be very difficult; therefore, we suggest having a bone-to-implant interface as wrapping as possible, with a deep groove extended on the external and partly lingual aspect of the mandible which would allow a better way to assure correct positioning.

It is important to remember that what seems to be easy and feasible on the computer might not be so in the operatory theatre, so eventual reference flanges and extension should be always carefully planned to follow the questions: can I check where this will go? Will I have blackholes where I would not be able to assess its correct positioning? This becomes especially true in the lingual aspect of the border of the mandible where detachment of the tissues is not always manageable and where vision is limited.

Another tip is to take advantage of any asperity or characteristic shape present on the surface of the bone; if these are correctly embedded in the implant, they can serve as useful references.

Regarding cases 6 to 9 in our practice, we observed satisfactory results using large, customised titanium and PEEK prostheses; the design and implantations were always feasible. The manufacturing of these prostheses, nevertheless, is extremely precise; therefore, we should always verify the patient’s clinical condition and especially the local conditions on the implant future site, a correct amount and quality of the surrounding tissue is deemed to be fundamental, and an insufficient amount of coverage or excessive shrinkage/reduced elasticity of the soft tissue would make implant insertion impossible or would lead to prosthesis exposure with subsequent complications. In those cases where local tissue conditions are not good enough, we suggest to properly prepare the implant site. At this stage, minimising complications is mandatory. Inadequately prepared or severely scarred tissues can lead to several complications.

In order to do so, in those cases with medium-moderate scar retraction, we prefer to use the “lipobed” technique with sequential lipofilling applications to be performed before prosthesisation. These infiltrations allow for the creation of a “chamber” within the fibrous tissues that make dissection easier, increasing the thickness and quality of the tissues that will serve as implant coverage; if scarring is severe, sometimes the use of fat free flaps is indicated.

Three-dimensional analysis and planning are a powerful tool for research and clinical applications. It sets the frame of reference to establish surgical goals after assessment and analysis. But relying on 3D which is mostly bone reconstruction only can also give the false impression that, once the implant is correctly designed to fit the alteration, the planning is done, but that is actually when the surgeon’s experience and knowledge of anatomy become crucial. Knowing the location of the main structures is important from a clinical point of view due to the risk of lesion to vessels, nerves, and ligaments during surgery carried out in the region.

## Conclusion

The planning of the implant mostly relies on hard tissue 3D reconstruction, but it should not be limited at what is immediately evident. A surgeon’s clinical experience should always guide the process, with knowledge of the patient’s anatomy and evaluation of the quality and of the soft tissue response being taken into consideration. The implant should always be tailored not only based on the bone defect and evaluations but also using the patient’s previewed and actual anatomy, evaluating eventual interferences and pitfalls.

## Data Availability

Data and material are available on demand.

## Abbreviations

2D: Bi-dimensional
3D: Three-dimensional
CAD/CAM: Computer-aided design/computer-aided manufacturing
CT: Computed tomography
PEEK: Polyetheretherketone
PSI(s): Patient-specific implant(s)
